# Surgical Fees at Charity Hospitals

**Published:** 1896-01-18

**Authors:** 


### Surgical Fees at Charity Hospitals.

It is easy to make out a case, as *Truth* does, against a surgeon who accepts a fee from a pay patient at a hospital, but rather than point at the surgeon in question we would arraign the system which is itself the origin of the difficulty. The recurrence of complaints of this nature is proof enough, if proof were needed, that our present hospital system is lacking in elasticity. So long as hospitals were looked upon as pest houses, places to be shunned and avoided at all hazards, things were simple enough. A man in those days must indeed have been hard hit before he would ask admission. But just in proportion as hospitals have improved and discomforts have been removed, so have those who are neither poor nor needy flocked in to share the good things offered so abundantly.

At present these better class patients occupy a position which is completely anomalous. On the strength of the contribution which they intend to make they think they pay, and sometimes are apt to put on airs in consequence. But the doctors, and all the people with whom they come in contact in the wards, know well enough that they do not pay them, and also see that by filling beds they are keeping poor men out. Nor do these better class patients conduce to the comfort or discipline of a ward. Being accustomed to little niceties they do not understand why they should not have them seeing they are "prepared to pay," and great is their indignation if the doctor lets fall a remark which shows that he looks upon them as charity patients, just in the same degree as the cobblers and street sweepers in adjoining beds!

The existing state of affairs is unsatisfactory in every way, and it is equally clear that there must be some reform. It is equally clear that the case is not met by allowing the doctor to haggle for his fees while the secretary makes his bargain in regard to the price to be paid for board and nursing. We shall soon have the nurses also making their arrangements with pay patients, and the dispenser asking special remuneration for the extra trouble caused by them. No. If hospitals are to receive patients who are not objects of charity some plan must be adopted by which their admission shall be made fair to all parties, and shall be in accordance with rules rather than in defiance of them, for it is quite certain that by hook or by crook the middle classes will squeeze into the hospitals now that treatment in them is so good and surgical treatment at home is so difficult and expensive.

There are three ways out of the difficulty: (1) The pay hospital ought, in large centres of population, to be the best plan; but the trust which the public have in the big hospitals is so great that when it comes to the point even well-to-do people often prefer them to any private institution. (2) Pay wards attached to general hospitals would meet the case of those who are prepared to pay a fair sum for their treatment—something over and above the mere expense to which the hospital may have been put in receiving them. (3) The system of receiving pay patients in general wards would meet the case of those who, while more or less objects of charity, are still able to pay something.

Both in regard to pay wards and pay patients it should be understood that a certain proportion of the fees received should go to the medical staff, a small proportion—perhaps a quarter—in the case of the contributions for treatment in the general wards; a larger proportion—perhaps a half—in the case of those treated in private wards. Just in proportion as the patients ceased to be objects of charity, so far as their keep was concerned, they would also become independent as in regard to their medical fees.

